# Predictive Value of Clinical and Questionnaire Based Screening Tools of Obstructive Sleep Apnea in Patients With Type 2 Diabetes Mellitus

**DOI:** 10.7759/cureus.18009

**Published:** 2021-09-16

**Authors:** Awais Muhammad Butt, Uneeba Syed, Adeel Arshad

**Affiliations:** 1 Department of Endocrinology and Metabolism, Services Hospital, Lahore, PAK

**Keywords:** epworth sleepiness scale, berlin questionaire, stop-bang questionnaire, obstructive sleep apnea, type 2 diabetes mellitus, screening tools, questionnaires

## Abstract

Background

Obstructive sleep apnea (OSA) is a major health problem for people with type 2 diabetes mellitus (DM2) and is associated with poorer glycemic control. Early detection is critical to proper management. In this study, we planned to assess and compare the diagnostic accuracy of various OSA screening tools in patients with DM2.

Methods

In this cross-sectional study, we consecutively recruited 58 patients with DM2, presenting to the endocrinology department of Services Hospital Lahore between February 2020 to March 2021. Along with demographic and anthropometric measurements, including BMI and neck circumference, participants answered the snoring, tiredness, observed apnea, high blood pressure - BMI, age, neck circumference, and gender (STOP-BANG) questionnaire, Berlin questionnaire, and Epworth sleepiness scale. All participants then underwent an overnight, level 3 polysomnography.

Results

The overall prevalence of OSA, diagnosed by overnight polysomnography, was 65.5% in type 2 diabeticDM2 patients. The STOP-BANG questionnaire had the highest sensitivity for mild, moderate, and severe OSA i.e., 84.2%, 90.3%, and 100% respectively. Berlin questionnaire also had 100% sensitivity for severe OSA and was most specific for mild and moderate OSA (70% and 63% respectively) whereas the Epworth sleepiness scale had the highest specificity of 53.3% for severe OSA.

Conclusion

This study shows that OSA is highly prevalent in DM2 patients in Pakistan. The STOP-BANG and Berlin questionnaire proved to be effective screening tools, especially for severe OSA. Results of our study should encourage the routine use of these questionnaires in clinical practice, to help in the early identification of OSA in diabetics.

## Introduction

Obstructive sleep apnea (OSA) is the intermittent, partial or complete, obstruction of the upper airway during sleep, leading to disturbed sleep and a plethora of functional and metabolic derangements, affecting multiple systems of the body, making it a major health problem [1]. The overall prevalence of OSA is on the rise, with a prevalence in moderate to severe OSA (≥15 events per hour) of 23.4% in women and 49.7% in men [2]. Reliable data regarding the prevalence of OSA in Pakistan, diagnosed by overnight polysomnography, is lacking till now [3]. On the basis of a questionnaire-based study, 10% of the Pakistani population was found to be at high risk for developing OSA [3].

Patients with type 2 diabetes mellitus (DM2) are at a greater risk to develop OSA as compared to general population. One study estimated the overall prevalence of OSA in DM2 patients, diagnosed by full polysomnography, to be approximately 71%, averaging the collective data from five studies, which included nearly 1200 participants with DM2, with prevalence ranging from 58% to 86% between the studies [4]. There seems to be a bidirectional relationship between diabetes mellitus and OSA, with both conditions perpetuating the risk of the other [5]. Intermittent hypoxemia and sleep fragmentation, caused by the recurrent upper airway obstruction due to OSA, lead to sympathetic system activation, hypothalamic-pituitary-adrenal axis alterations, adipokine disturbances, systemic inflammation, and oxidative stress, resulting in impaired glycemic control and greater insulin resistance. On the other hand, autonomic neuropathy, insulin resistance, leptin resistance, and overall oxidative stress, which are consequences of diabetes mellitus, may alter the neuronal and mechanical control of the upper airway muscles, leading to easy collapsibility of these muscles during sleep, causing OSA [5].

OSA in patients with DM2 is associated with poorer glycemic control and greater hemoglobin A1c (HbA1c) levels [6]. There is evidence that early treatment of OSA, such as continuous positive airway pressure (CPAP), may lead to better glycemic control in patients with DM2 by improving insulin resistance, with a possibly greater benefit in patients with more severe OSA and poorer baseline glycemic control, provided there is better compliance and longer duration of CPAP treatment [7]. Early diagnosis is the key.

According to the International classification of sleep disorders-third edition (ICSD-3), in a diabetic patient, the finding of five or more predominantly obstructive respiratory events, as observed by polysomnography, is sufficient for diagnosis of OSA even in the absence of typical signs and symptoms of OSA [8]. However, there is a concern that OSA is not properly looked for in the primary care setting leading to gross underdiagnosis of OSA in DM2 [9].

Overnight polysomnography is required to confirm the diagnosis of OSA. However, polysomnography is time-consuming, expensive, and not easily available everywhere. Several clinical predictors and questionnaires are also used to screen for or diagnose OSA, some of which include BMI [10], neck circumference [11], Berlin questionnaire [12], Epworth sleepiness scale [13], and the snoring, tiredness, observed apnea, high blood pressure - BMI, age, neck circumference, and gender (STOP-BANG) questionnaire [14], but these have variable sensitivity and specificity, and polysomnography remains the gold standard diagnostic test for OSA [15].

In a resource-depleted country like Pakistan, the use and availability of polysomnography are very restricted. Even research data regarding OSA confirmed by polysomnography is limited. To the best of our knowledge, only one study has been done in Pakistan in which OSA was diagnosed with polysomnography involving only 30 general patients [16]. The few other studies involving diagnosis and prevalence of OSA were based on questionnaires only. There is a need for more research on OSA in Pakistan, especially in diabetic patients, whose number is increasing day by day. There is also a need for developing cost-effective and efficient diagnostic screening tools for OSA to help in early and timely referral for formal sleep studies, leading to earlier and better management of OSA, prevention of its debilitating complications, and cutting costs.

In this study involving adult patients with DM2, we aimed to assess and compare the predictive value of clinical and questionnaire-based screening tools including BMI, neck circumference, STOP-BANG questionnaire, Berlin questionnaire, and Epworth sleepiness scale in diagnosing OSA, confirmed by overnight polysomnography.

## Materials and methods

This was a cross-sectional study, in which we included 58 consecutive patients with DM2, without a previous diagnosis of OSA, admitted to the endocrinology ward of the Services Hospital Lahore, between February 2020 and May 2021. The study was approved by the Institutional Review Board of Services Institute of Medical Sciences/ Service Hospital Lahore (Ref No. IRB/2020/637/SIMS).

Those included were ≥ 18 years old, clinically diagnosed cases of DM2 (based on history and laboratory values including either fasting blood glucose >126 mg/dl or HbA1c > 6.5% or using anti-diabetic medication) and willing to participate in the study. We excluded those who were younger than 18 years, having type 1 diabetes mellitus, unstable cardiopulmonary disease, sepsis due to any cause, history of acute (within three weeks) or chronic respiratory tract infection, upper respiratory tract malignancy or mass, pregnancy, history of sedative use, sleep duration of fewer than four hours, and those unable to lie supine. Due to the emergence of the coronavirus disease 2019 (Covid-19) pandemic during the study, we added the exclusion criteria of previous history of Covid-19 pneumonia requiring hospital admission. All participants had to get a Covid-19 polymerase chain reaction (PCR) and chest x-ray at the time of admission to the hospital (as per institutional policy) and only those patients, who had a negative Covid-19 PCR and normal chest x-ray, were included in the study. Strict adherence to the institutional protocols for personal protective equipment (PPE) use by both patients and researchers was ensured. Patient contact was minimized as the overnight polysomnography was unobserved.

Data collection

Informed consent was taken from all the participants. History, physical examination, routine demographic data, and anthropometric measurements including height, weight, BMI, neck, waist, and hip circumference were noted. Routine investigations for diabetic patients, as per department protocol was done. All participants were screened using Interviewer-administered, validated Urdu versions of the STOP-BANG questionnaire [17], Berlin questionnaire [3], and Epworth sleepiness scale [18]. All participants then underwent an overnight, level 3 polysomnography (unobserved), using Alice PDx portable sleep diagnostic system (Philips Respironics, Murrysville, United States), in the endocrinology ward of Services Hospital Lahore. The polysomnography recorded chest and abdominal respiratory movements, nasal pressure and oral thermistor, oxygen saturation, heart rate, and body position.

Operational definitions

Apnea Hypopnea Index (AHI)

It is the number of events of hypopnea or apnea per hour, which was determined by an overnight, level 3 polysomnography (unobserved), using the Alice PDx sleep diagnostic system. It was calculated by the automated scoring software Sleepware G3 with Somnolyzer 24x7 (Philips Respironics, Murrysville, United States), based on the American Association of Sleep Medicine (AASM) scoring manual. Minimum four hours sleep study duration was mandatory.

Obstructive Sleep Apnea (OSA)

OSA was defined as an apnea-hypopnea index (AHI) of ≥ 5. Mild OSA was defined as an AHI of 5 - 14, Moderate OSA as AHI of 15 - 29, and Severe OSA as AHI ≥30 OSA [19].

Body Mass Index 

BMI was calculated by dividing weight (kg) by the square of height (m). Weight was measured in light clothing. Height was measured without shoes. A BMI of 30 kg/m2 or more, was considered as high risk for OSA [19].

Neck Circumference

Neck circumference was measured at the middle of the neck, between the mid-cervical spine and mid-anterior neck (Cricothyroid membrane), in centimeters, using a non-stretchable plastic measuring tape, with the patient sitting and looking forward. A neck circumference of 43 cm (17 in) or more in males and 41.5 cm (16 in) or more in females, was considered as high risk for OSA [19].

STOP-BANG Questionnaire

 A validated Urdu version of the STOP-BANG questionnaire was used [17] (See Appendix). It consists of eight dichotomous questions with yes or no answers. Each positive reply (Yes) carries a score of 1. A total score of three or more than three was considered as high risk for OSA, and <3 was considered as low risk for OSA [20].

Berlin Questionnaire

A validated Urdu version of the Berlin questionnaire was used [3] (See Appendix). It consists of 10 questions, divided into three categories, regarding snoring (category 1), daytime sleepiness (category 2), hypertension, and BMI (Category 3). The overall score was determined from the responses to the three categories. Scores from the first and second categories were considered positive if the responses indicated frequent symptoms (>3-4 times/week), whereas the score from the third category was considered positive if there was a history of hypertension or a BMI >30 kg/m2. Patients were considered to be at high risk for OSA if two or more categories had a positive score, and low risk for OSA if only one or no category had a positive score [12].

Epworth Sleepiness Scale

A validated Urdu version of the Epworth sleepiness scale was used [18] (See Appendix)​​​​​​. It consisted of eight questions related to the tendency to fall asleep in various situations. The answer to each question was on a scale of 0 to 3, depending on the likelihood of falling asleep. This scale of zero to three was also the score of that question, with a total score of 24. A score of >10 was considered as high risk for OSA and less than 10 as low risk for OSA [21].

Data analysis

Data were analyzed using IBM SPSS Statistics for Windows, Version 25.0 (Released 2017, IBM Corp., Armonk, NY). For quantitative data, means with standard deviation were calculated, whereas percentages were calculated for categorical data. Chi-square test and independent samples t-test were used to detect the statistical difference of categorical and quantitative variables, respectively, between two groups. Sensitivity, specificity, positive predictive value (PPV), and negative predictive value (NPV) of each screening tool were determined, using the AHI-based criteria from polysomnography as the gold standard for diagnosis of OSA. Receivers operator characteristics area under the curve (ROC-AUC) was calculated for each screening tool to assess its discriminating potential and was compared with the others.

## Results

There were 58 participants in the study, 62.1% (36) being male and 37.9% (22) female. The mean age of the study participants was 49.84 years (±6.73) and the mean BMI was 32.58 (±3.35). The main characteristics of the study population are provided in Table 1.

**Table 1:** Main characteristics of the study population Values given as mean with standard deviation for continuous variables, and number with percentage for categorical variables SD: standard deviation; FBG: fasting blood glucose; IHD: ischemic heart disease; CKD: chronic kidney disease; AHI: apnea-hypopnea index; STOP-BANG: snoring, tiredness, observed apnea, high blood pressure - BMI, age, neck circumference, and gender

Characteristics		Mean / N	SD / %	Characteristics	Mean / N	SD / %
Gender	Males	36	62.1	Diabetes Duration (years)	4.53	2.90
	Females	22	37.9	FBG (mg/dl)	180.74	58.13
Age (Years)		49.84	6.73	HbA1c (%)	9.99	1.76
Weight (kg)		87.02	12.75	Hypertension	29	50
Height (cm)		163.27	9.30	IHD	7	12.1
BMI (kg/m^2^)		32.58	3.35	CKD	8	13.8
Waist Circumference (cm)		111.33	8.47	AHI (Events/hour)	17.95	13.16
Neck Circumference (cm)	Male	43.39	2.99	STOP-BANG Score	3.95	1.85
	Female	39.36	2.92	Epworth Sleepiness Score	8.79	4.61

Of the participants, 53.4% (31) and 69% (42) were at high risk for OSA according to the cut-offs for neck circumference and BMI, respectively, whereas 74.1% (43), 62.1% (36), and 44.8% (26) participants fell in the high-risk category according to the STOP-BANG questionnaire, Berlin questionnaire, and Epworth sleepiness scale, respectively.

Based on results of overnight polysomnography, the prevalence of OSA in our sample of DM2 patients was 65.5% (38), out of which 12.1 % (7) had mild OSA (AHI 5-14), 31% (18) had moderate OSA (AHI 15-29) and 22.4% (13) had severe OSA (AHI > 30). The prevalence of OSA in males was 61.1% (22) and in females, it was 72.7% (16).

Those with OSA had significantly higher mean BMI (33.33 ±3.62 vs 31.16 ±2.23, p=.007), HbA1c (10.35 ±1.75 vs 9.31 ±1.59), and STOP-BANG questionnaire score (4.63 ±1.78 vs 2.65 ±1.18, p=<.001) compared to those who did not have OSA. The comparison between the two groups is given in Table 2.

**Table 2:** Comparison between the characteristics of the OSA and non-OSA groups, based on overnight polysomnography Values given as mean with standard deviation for continuous variables, and number with percentage for categorical variables. Difference between means estimated using independent samples t-test. Difference between frequency/percentage estimated using chi-square test. *Significant difference between groups OSA: obstructive sleep apnea; FBG: fasting blood glucose; HTN: hypertension; IHD: ischemic heart disease; CKD: chronic kidney disease; AHI: apnea-hypopnea index; STOP-BANG: snoring, tiredness, observed apnea, high blood pressure - BMI, age, neck circumference, and gender

Characteristic		No OSA (n=20)	OSA (n=38)	p-value
Gender	Male	14 (38.9%)	22 (61.1%)	.182
	Female*	6 (27.3%)	16 (72.7%)	.033
Age (years)		49.70(±6.48)	49.92(±6.94)	.907
Height (cm)		162.81(±10.07)	163.51(±9.00)	.787
Weight (kg)		82.63 (±9.67)	89.33 (±13.66)	.057
Waist (cm)		109.90 (±6.94)	112.08 (±9.18)	.357
FBG (mg/dl)		168.20 (±46.68)	187.34 (±62.90)	.237
HbA1c (%)*		9.31 (±1.59)	10.35 (±1.75)	.031
HTN		7 (35%)	22 (57.9%)	.097
IHD		1 (5%)	6 (15.8%)	.403
CKD		2 (10%)	6 (15.8%)	.701
BMI* (kg/m^2^)		31.16(±2.23)	33.33(±3.62)	.007
Neck circumference (cm)	Males	42.29 (±3.31)	44.09 (±2.60)	.077
	Females	39.50 (±2.88)	39.31 (±3.03)	.897
STOP-BANG Score*		2.65 (±1.18)	4.63 (±1.78)	< .001>
Epworth Sleepiness Score		9.0 (±5.23)	8.68 (±4.32)	.807
AHI* (events/hour)		4.26(±0.59)	25.15(±10.58)	< .001>

Sensitivity, specificity, positive predictive value (PPV), and negative predictive value (NPV)

For AHI ≥5 and ≥15, the STOP-BANG questionnaire had the highest sensitivity i.e., 84.2% and 90.3% respectively, whereas the Berlin questionnaire had the highest specificity i.e., 70% and 63% respectively. For AHI ≥30, both the STOP-BANG questionnaire and the Berlin questionnaire had 100% sensitivity, while the Epworth sleepiness scale had the highest specificity at 53.3%. The sensitivity, specificity, PPV, and NPV of the various screening tools, for different AHI cut-offs, are shown in Table 3.

**Table 3:** Sensitivity, specificity, PPV, and NPV of screening tools, according to different AHI cut-offs for diagnosis of obstructive sleep apnea in type 2 diabetes patients PPV: positive predictive value; NPV: negative predictive value; AHI: apnea-hypopnea index; STOP-BANG: snoring, tiredness, observed apnea, high blood pressure - BMI, age, neck circumference, and gender

Screening Tool	Sensitivity	Specificity	PPV	NPV
AHI ≥ 5				
STOP-BANG questionnaire	84.2	45.0	74.4	60
Berlin questionnaire	78.9	70.0	83.3	63.6
Epworth sleepiness scale	44.7	55.0	65.4	34.4
Neck circumference	60.5	60.0	74.2	44.4
BMI	73.7	40	70	44.4
AHI ≥ 15				
STOP-BANG questionnaire	90.3	44.4	65.1	80.0
Berlin questionnaire	83.9	63.0	72.2	77.3
Epworth sleepiness scale	45.2	55.6	53.8	46.9
NC	61.3	55.6	61.3	55.6
BMI	77.4	40.7	60.0	61.1
AHI ≥ 30				
STOP-BANG questionnaire	100	33.3	30.2	100
Berlin questionnaire	100	48.9	36.1	100
Epworth sleepiness scale	38.5	53.3	19.2	75.0
Neck circumference	69.2	51.1	29.0	85.2
BMI	76.9	33.3	25.0	83.3

On ROC-AUC analysis, the STOP-BANG questionnaire had the largest AUC (0.809, 0.856, and 0.900 for AHI ≥5, ≥15, and ≥30 respectively) compared to Epworth sleepiness scale, neck circumference, and BMI, as shown in Table 4. Berlin questionnaire was not included in the analysis as it was reported as a dichotomous variable.

**Table 4:** ROC-AUC analysis of screening tools according to different AHI cut-offs for diagnosis of OSA in type 2 diabetes patients *p=<0.05, **p=<0.01, ***p=<0.001 ROC-AUC: Receivers operating curve – area under the curve; AUC: area under the curve; AHI: apnea-hypopnea index; OSA: obstructive sleep apnea; STOP-BANG: snoring, tiredness, observed apnea, high blood pressure - BMI, age, neck circumference, and gender

Screening Tool	AUC (95% CI)		
	AHI >5	AHI > 15	AHI > 30
STOP-BANG questionnaire	0.809*** (0.701-0.916)	0.856*** (0.757-0.955)	0.900*** (0.805-0.995)
Epworth sleepiness scale	0.483 (0.315-0.651)	0.466 (0.314-0.618)	0.449 (0.283-0.615)
BMI	0.671* (0.530-0.812)	0.675* (0.532-0.818)	0.744 ** (0.554-0.933)
Neck circumference	0.561 (0.405-0.716)	0.554 (0.403-0.705)	0.595 (0.431-0.759)

## Discussion

OSA is becoming a major health problem globally, especially in people with DM2, in whom it is associated with worsening glycemic control. Our results, based on AHI criteria laid down by the AASM for diagnosis of OSA in diabetic patients and assessed by overnight polysomnography, show that almost two-thirds (65.5%) of the DM2 patients in our population have some degree of OSA. Out of these, nearly one-third have severe OSA (AHI ≥ 30). This large prevalence of OSA in the DM2 population has also been reported in previous studies, with prevalence ranging from 57.7% to 86% [4], with almost half of these having moderate to severe OSA, which underscores the significance of this condition in this population.

In our study we assessed and compared the diagnostic efficacy of the STOP-BANG questionnaire, Berlin questionnaire, Epworth sleepiness scale, BMI, and neck circumference in detecting OSA. In terms of sensitivity, we found the STOP-BANG questionnaire to be the most sensitive screening tool for mild and moderate OSA, followed closely by the Berlin questionnaire. For severe OSA, both these questionnaires had 100% sensitivity. BMI and neck circumference had much lower sensitivity while Epworth sleepiness scale turned out to be the least sensitive screening tool. Both the STOP-BANG questionnaire and the Berlin questionnaire have been shown to be valid and effective screening tools for OSA in general population [12,14,22] as well as DM2 patients [23,24] but the STOP-BANG questionnaire had greater sensitivity as compared to the Berlin questionnaire, especially for mild and moderate OSA. Kim et al. found the STOP-BANG questionnaire to have 97% and 98% sensitivity for AHI ≥5 and AHI ≥15 respectively, whereas the Berlin questionnaire had a sensitivity of 71.5% and 75.5% for the same [25]. Westlake et al. found the STOP-BANG questionnaire to have higher sensitivity for mild, moderate and severe OSA, compared to the Berlin questionnaire [26]. A meta-analysis of 108 studies reported similar results [27].

The Berlin questionnaire showed the greater specificity for mild and moderate OSA compared to other screening tools, especially the STOP-BANG questionnaire, which is similar to the previous findings of Kim et al. [25] and Westlake et al. [26]; however, Epworth sleepiness scale had the greatest specificity for severe OSA compared to others. Silva et al., in their study of 4770 participants of the Sleep Heart Health Study population, found the Epworth sleepiness scale to have a specificity of 71.4% for moderate to severe OSA, compared to a specificity of 43.3% of the STOP-BANG questionnaire [22]. Pataka et al., in their comparison of the Epworth sleepiness scale, STOP-BANG questionnaire, and Berlin questionnaire in DM2 patients, found the Epworth sleepiness scale to have the highest specificity for moderate to severe OSA [23].

The higher sensitivity of the STOP-BANG questionnaire and Berlin questionnaire may be due to the fact that both these questionnaires rely mainly on the presence and characterization of snoring, which is the most common symptom of OSA [28]. They also incorporate BMI and neck circumference cut-offs in to the scoring, which further lessens the chances of a false negative result. Whereas the Epworth sleepiness scale is basically a sleepiness score and significantly marked somnolence may be a late consequence of OSA, signifying severe disease, making it more specific for severe disease.

Further supporting the superiority of the STOP-BANG questionnaire, our ROC-AUC analysis of STOP-BANG questionnaire score, Epworth sleepiness scale score, BMI, and neck circumference as continuous variables, showed that the STOP-BANG questionnaire had the greatest discriminating power for mild, moderate, and severe OSA. These AUC values are very similar to those reported by Teng et. al. [24], in DM2.

In our study, DM2 patients with OSA had a significantly higher mean HbA1c as compared to those not having OSA. This inverse relationship between OSA and glycemic control is well established in previous studies [5,29], which again emphasizes the significant impact of OSA on diabetic patients and the imperative need to diagnose and treat these patients earlier.

The use of level 3 polysomnography as the gold standard test to diagnose OSA in DM2 was the major strength of our study, which was the first of its kind in Pakistan. The main limitation of our study was the small sample size, mainly due to Covid-19 related restrictions on admissions during the study period. Small sample size can affect the precision and reliability of the study results. Since the prevalence of OSA in diabetics is high (around 70% in our study) and we were mainly concerned with the sensitivity of the screening tools, a minimum sample size of 44 was required to achieve a minimum power of 80% for an expected sensitivity level of at least 90% and a significance level of 0.05 [30]. On the other hand, the results for specificity may be affected by this small sample size. Another limitation was the lack of a control group of age and gender matched non-diabetic population. However, the prevalence of OSA in diabetics was similar to that reported in other studies [4]. Home-based polysomnography facilities are very expensive and difficult to acquire in our setup, which preclude the testing in outdoor patients. Thus, we strongly feel that further research with a larger sample size and a wider spectrum of glycemic control is warranted in our population.

## Conclusions

In conclusion, OSA is a serious condition that is widely prevalent in the DM2 population of Pakistan according to our results, making it an important diagnostic and treatment target in this population. The early diagnosis of OSA is vital for preventing the deleterious effects of this condition in diabetic patients as it provides the opportunity for early intervention and possible treatment. Effective screening tools such as the STOP-BANG questionnaire and Berlin questionnaire will prove helpful in efficiently streamlining the resource allocation and efforts towards the diagnosis of OSA, as the gold standard test of polysomnography remains quite resource intensive.

## Figures and Tables

**Figure 1:**
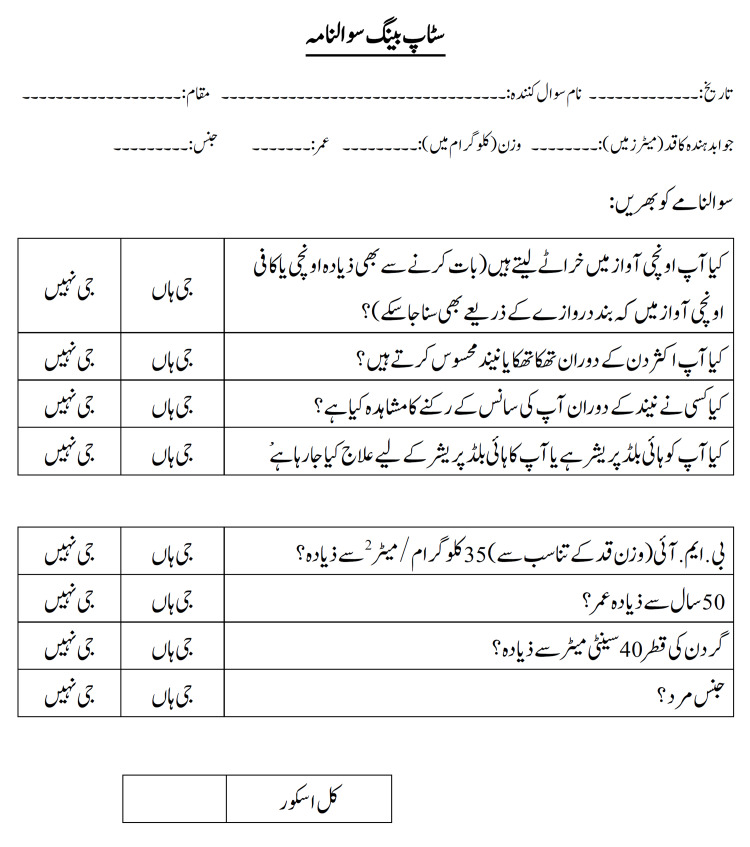
Urdu version of STOP-BANG questionnaire Adapted from Ali et al. 2016 [17] STOP-BANG: snoring, tiredness, observed apnea, high blood pressure - BMI, age, neck circumference, and gender

**Figure 2:**
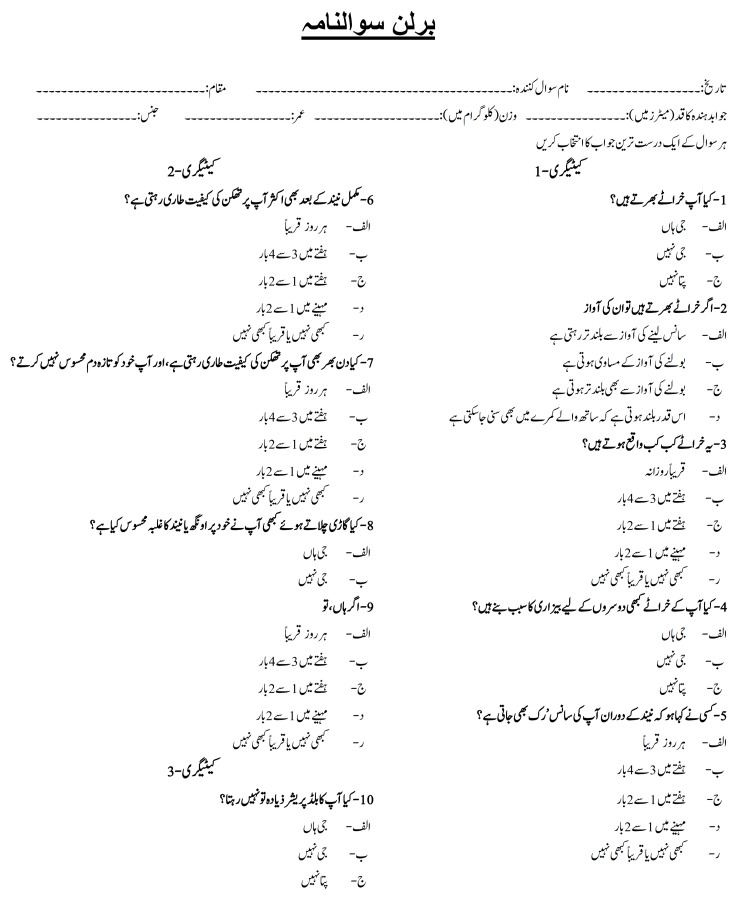
Urdu version of Berlin questionnaire Adapted from Taj et al. 2008 [3]

**Figure 3:**
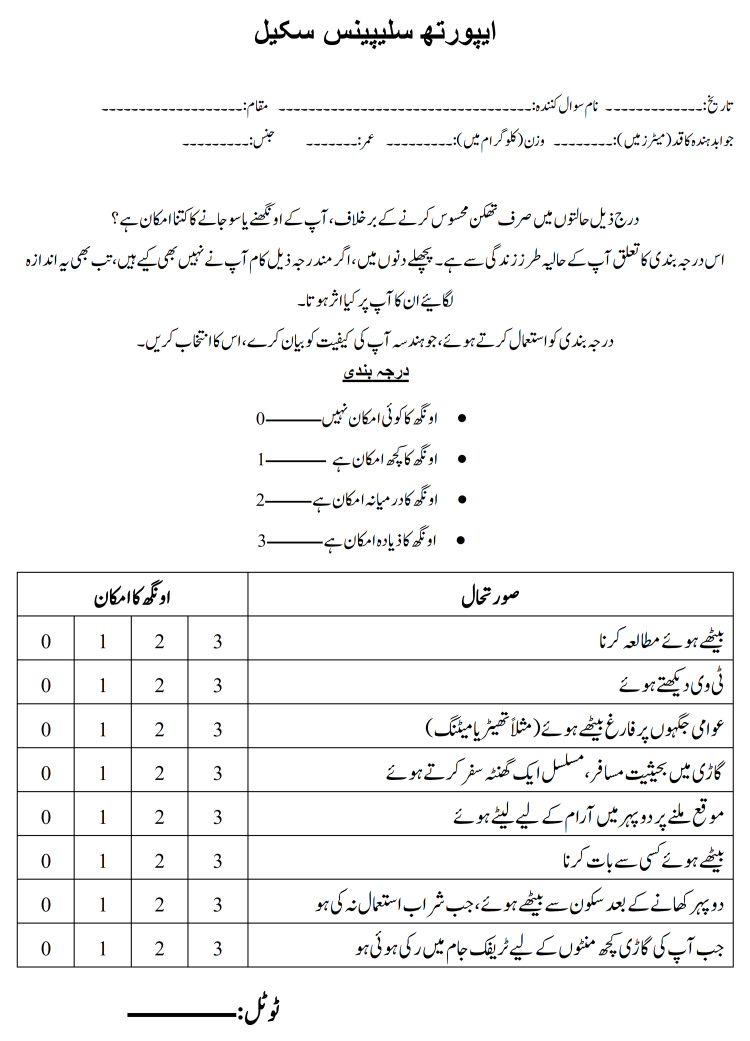
Urdu version of Epworth sleepiness scale Adapted from Surani et al. 2012 [18]
